# Expression of fatty acid and lipid biosynthetic genes in developing endosperm of *Jatropha curcas*

**DOI:** 10.1186/1754-6834-5-47

**Published:** 2012-07-18

**Authors:** Keyu Gu, Chengxin Yi, Dongsheng Tian, Jatinder Singh Sangha, Yan Hong, Zhongchao Yin

**Affiliations:** 1 Temasek Life Sciences Laboratory, 1 Research Link, National University of Singapore, Singapore, 117604, Republic of Singapore; 2 JOil (S) Private Limited, 1 Research Link, National University of Singapore, Singapore, 117604, Republic of Singapore; 3 Department of Biological Sciences, 14 Science Drive, National University of Singapore, Singapore, 117543, Republic of Singapore

**Keywords:** Fatty acid and lipid biosynthesis, *Jatropha curcas*, Endosperm development, Oil body, Gene expression, Real-time PCR

## Abstract

**Background:**

Temporal and spatial expression of fatty acid and lipid biosynthetic genes are associated with the accumulation of storage lipids in the seeds of oil plants. In jatropha (*Jatropha curcas* L.), a potential biofuel plant, the storage lipids are mainly synthesized and accumulated in the endosperm of seeds. Although the fatty acid and lipid biosynthetic genes in jatropha have been identified, the expression of these genes at different developing stages of endosperm has not been systemically investigated.

**Results:**

Transmission electron microscopy study revealed that the oil body formation in developing endosperm of jatropha seeds initially appeared at 28 days after fertilization (DAF), was actively developed at 42 DAF and reached to the maximum number and size at 56 DAF. Sixty-eight genes that encode enzymes, proteins or their subunits involved in fatty acid and lipid biosynthesis were identified from a normalized cDNA library of jatropha developing endosperm. Gene expression with quantitative reverse-transcription polymerase chain reaction analysis demonstrated that the 68 genes could be collectively grouped into five categories based on the patterns of relative expression of the genes during endosperm development. Category I has 47 genes and they displayed a bell-shaped expression pattern with the peak expression at 28 or 42 DAF, but low expression at 14 and 56 DAF. Category II contains 8 genes and expression of the 8 genes was constantly increased from 14 to 56 DAF. Category III comprises of 2 genes and both genes were constitutively expressed throughout endosperm development. Category IV has 9 genes and they showed a high expression at 14 and 28 DAF, but a decreased expression from 42 to 56 DAF. Category V consists of 2 genes and both genes showed a medium expression at 14 DAF, the lowest expression at 28 or 42 DAF, and the highest expression at 56 DAF. In addition, genes encoding enzymes or proteins with similar function were differentially expressed during endosperm development.

**Conclusion:**

The formation of oil bodies in jatropha endosperm is developmentally regulated. The expression of the majority of fatty acid and lipid biosynthetic genes is highly consistent with the development of oil bodies and endosperm in jatropha seeds, while the genes encoding enzymes with similar function may be differentially expressed during endosperm development. These results not only provide the initial information on spatial and temporal expression of fatty acid and lipid biosynthetic genes in jatropha developing endosperm, but are also valuable to identify the rate-limiting genes for storage lipid biosynthesis and accumulation during seed development.

## Background

Plant storage lipids are a major food source. They also provide a vast range of renewable industrial and pharmaceutical products. They may be accumulated in one or both of the main types of seed tissue, the embryo or endosperm. Plant lipids are synthesized via a complex series of pathways in which many fatty acid and lipid biosynthetic enzymes and proteins are involved. Although the pathways for fatty acid and lipid biosynthesis are well understood, little is known about how plants regulate the varying amounts and types of lipids produced in different tissues or organs, especially in seeds.

The initial step to address this issue may be to investigate the spatial and temporal expression of fatty acid and lipid biosynthetic genes. Ruuska et al. (2002) used cDNA microarrays to compare gene expression during Arabidopsis seed development between wild-type and a mutant *wrinkled1* (*wri1*) seeds that have an 80 % reduction in oil. One of their significant findings was that a number of genes encoding core fatty acid synthesis enzymes displayed a bell-shaped pattern of expression between 5 and 13 days after flowering, a period preceding and including the major accumulation of storage oils and proteins [1]. O’Hara et al. (2002) determined the spatial and temporal expression of fatty acid and lipid biosynthetic genes during embryogenesis in *Brassica napus* (*B. napus*) and found that most of the fatty acid and lipid biosynthetic genes were expressed at constant molar ratios but different absolute levels during embryogenesis [2]. In another study, Dong et al. (2004) demonstrated that 104 genes were differentially expressed in *B. napus* seeds at 15 days after fertilization (DAF), but this number was reduced to 63 at 25 DAF [3]. Niu et al. (2009) performed cDNA chip hybridization (>8000 EST clones from *B. napus* seeds) and revealed that the crucial stage for the transition of seed-to-sink tissue was 17–21 DAF, whereas fatty acid biosynthesis-related genes were highly expressed primarily at 21 DAF [4].

Jatropha (*Jatropha curcas* L.), belonging to the Euphorbiaceae family, is a shrub that normally thrives in tropical and subtropical countries. Jatropha seeds contain high amount of oil that accumulate mainly in the endosperm of seeds. Jatropha is considered to be a potential biofuel plant as the fatty acid and lipid profile of jatropha oil is highly suitable for use as biodiesel [5]. Recently, jatropha has garnered immense attention in biological studies, in particular the genes that are involved in fatty acid and lipid biosynthetic pathways [6-11]. A recent study reported the identification of 7,009 unigenes from a normalized cDNA library of jatropha developing seeds and, of which, 17 genes encoding enzymes for fatty acid and lipid biosynthesis were further characterized for gene expression by quantitative reverse-transcription polymerase chain reaction (qRT-PCR) [8]. More recently, Xu et al. (2011) investigated temporal expression profiles of 21 lipid genes in developing jatropha seeds and found that 17 genes displayed elevated expression [12]. Although the genome size of jatropha is relatively small (C = 416 Mb) [13], systemic expression profiles of genes involved in fatty acid and lipid biosynthesis during jatropha seed development have yet to be elucidated. Therefore, identification of genes involved in fatty acid and lipid biosynthesis and characterization of their expression patterns are two essential prerequisites to understand genetic factors regulating storage lipid biosynthesis in jatropha seeds.

In this study, we examined oil body development in endosperm of jatropha developing seeds, identified fatty acid and lipid biosynthetic genes from a normalized cDNA library of jatropha developing endosperm and determined their expression patterns in developing seeds. Our results yield abundant information on jatropha genes that are involved in storage lipid biosynthesis and their expression patterns during seed development, which provide guidelines on breeding and genetic engineering of jatropha for high storage lipids.

## Results

### Oil body development in jatropha developing endosperm

**Figure 1:**
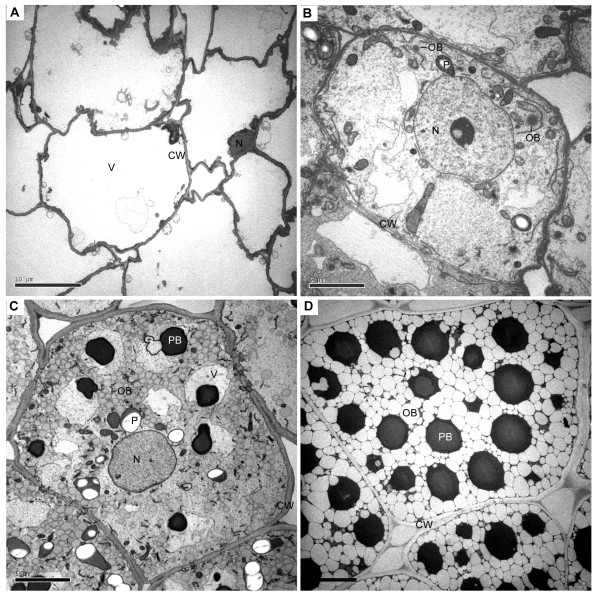
**Oil body and endosperm development in jatropha seeds.** Endosperm of jatropha seeds at 14 (**A**), 28 (**B**), 42 (**C**) and 56 (**D**) days after fertilization was examined with transmission electron microscopy. Two oil bodies (OB) are indicated. CW, cell wall; N, nucleus; OB, oil body; P, plastid; PB, protein body; V, vacuole.

In order to investigate storage lipid accumulation, especially the oil body development in endosperm cells of jatropha seeds, developing endosperm at 14, 28, 42 and 56 DAF was subjected to transmission electron microscopy (TEM) study. At 14 DAF, no oil body could be found in endosperm cells and most of the space was occupied by a huge central vacuole (Figure 1A). The endosperm cells started to develop oil bodies at 28 DAF (Figure 1B). At this stage, only several dark electron-dense oil bodies were observed in each cell (Figure 1B). Oil bodies were actively synthesized at 42 DAF (Figure 1C). The electron density of oil bodies at 42 DAF were lighter than that of oil bodies at 28 DAF (Figure 1C). Protein bodies were also synthesized at 42 DAF (Figure 1C). At 56 DAF, most of the space in endosperm cells was occupied by oil bodies and protein bodies (Figure 1D). The oil bodies at 56 DAF were also the lightest in term of electron density among oil bodies at 28, 42 and 56 DAF (Figure 1B to 1D). The difference in electron density of oil bodies may reflect difference of lipids and other components in the oil bodies. The TEM studies demonstrated that biosynthesis and accumulation of storage lipids in endosperm cells of jatropha seeds were developmentally regulated.

### Patterns of relative expression of fatty acid and lipid biosynthetic genes during endosperm development

**Figure 2:**
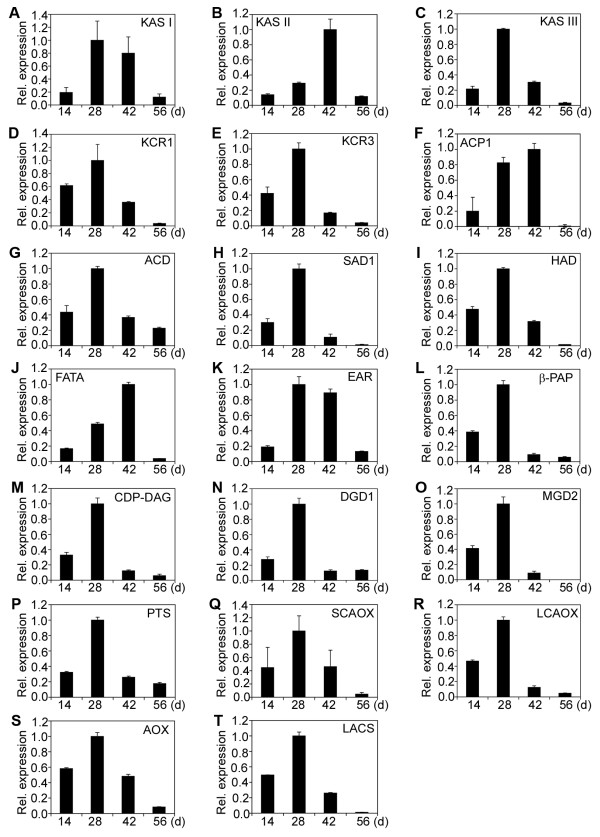
**Relative expression of fatty acid and lipid biosynthetic genes (I).** (**A**) Ketoacyl ACP synthase I (KAS I). (**B**) Ketoacyl ACP synthase II (KAS II). (**C**) Ketoacyl ACP synthase III (KAS III). (**D**) 3-ketoacyl-CoA reductase 1 (KCR1). (**E**) 3-ketoacyl-CoA reductase 3 (KCR3). (**F**) Plastial isoform of acyl carrier protein 1 (ACP1). (**G**) Acyl-CoA dehydrogenase (ACD). (**H**) Semialdehyde decarboxylase 1 (SAD1). (**I**) Hydroxyacyl-ACP dehydratase (HAD). (**J**) Acyl-ACP thioesterase (FATA). (**K**) Enoyl-ACP reductase (EAR). (**L**) Phosphatidic acid phosphatase β (β-PAP). (**M**) CDP-diacylglycerol synthase (CDP-DAG). (**N**) Digalactosyldiacylglycerol synthase 1 (DGD1). (**O**) Monogalactosyldiacylglycerol synthase 2 (MGD2). (**P**) Phosphatidylinositol synthase (PTS). (**Q**) Short-chain acyl-CoA oxidase (SCAOX). (**R**) Long-chain acyl-CoA oxidase (LCAOX). (**S**) Acyl-CoA oxidase (AOX). (**T**) Long-chain acyl-CoA synthase (LACS). The gene transcripts were measured by qRT-PCR. Results are shown as the relative expression of genes at different developmental stages by comparing to itself at the highest expression, which was set as “1”. The experiments were performed in triplicate and the data are presented as means ± SD.

**Figure 3:**
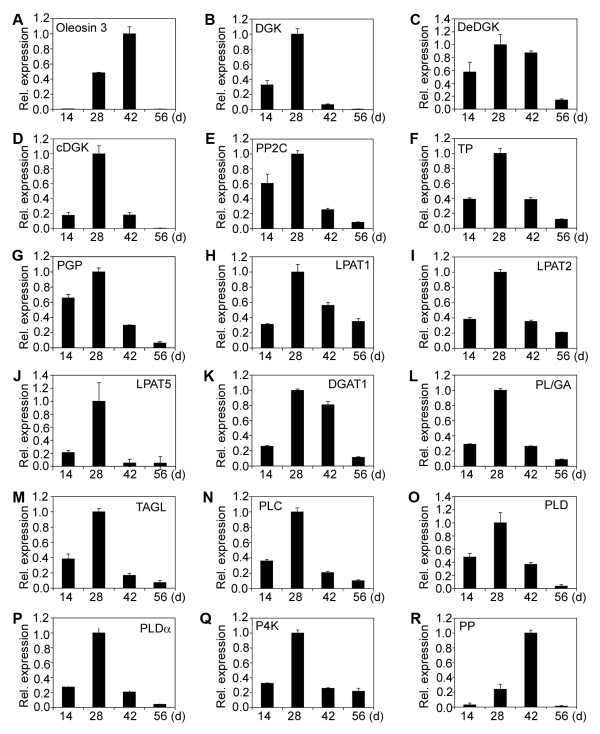
**Relative expression of fatty acid and lipid biosynthetic genes (II).** (**A**) Oleosin 3. (**B**) Diacylglycerol kinase (DGK). (**C**) D-erythro-sphingosine kinase/diacylglycerol kinase (DeDGK). (**D**) Calmodulin-binding diacylglycerol kinase (cDGK). (**E**) Protein phosphatase 2 C (PP2C). (**F**) Tyrosine phosphatase (TP). (**G**) Phosphoglycolate phosphatase (PGP). (**H**) Lysophosphatidyl acyltransferase 1 (LPAT1). (**I**) Lysophosphatidyl acyltransferase 2 (LPAT2). (**J**) Lysophosphatidyl acyltransferase 5 (LPAT5). (**K**) Diacylglycerol acyltransferase (DGAT1). (**L**) Phospholipids/glycerol acyltransferase (PL/GA). (**M**) Triacylglycerol lipase (TAGL). (**N**) Phospholipase C (PLC). (**O**) Phospholipase D (PLD). (**P**) Phospholipase D α (PLDα). (**Q**) Phosphatidylinositol 4-kinase (P4K). (**R**) Putative phosphatase (PP). The gene transcripts were measured by qRT-PCR. Results are shown as the relative expression of genes at different developmental stages by comparing to itself at the highest expression, which was set as “1”. The experiments were performed in triplicate and the data are presented as means ± SD.

**Figure 4:**
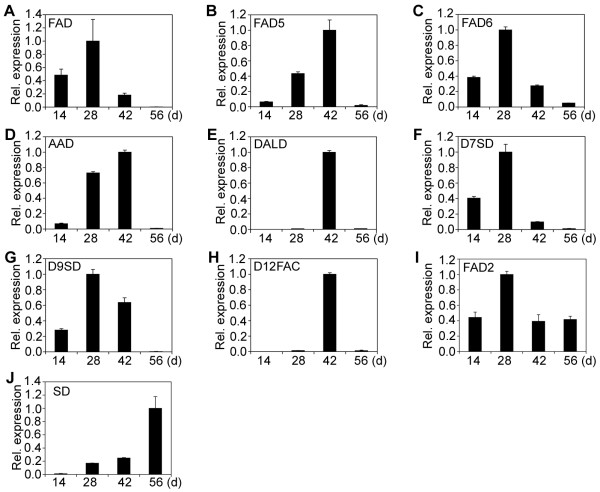
**Relative expression of desaturase genes.** (**A**) Fatty acid desaturase (FAD). (**B**) Fatty acid desaturase 5 (FAD5). (**C**) Fatty acid desaturase 6 (FAD6). (**D**) Acyl-ACP desaturase (AAD). (**E**) δ-12-acyl-lipid desaturase (DALD). (**F**) δ-7-C-5 sterol desaturase (D7SD). (**G**) δ-9-stearoyl-acyl carrier protein desaturase (D9SD). (**H**) δ-12 fatty acid conjugase (D12FAC). (**I**) Fatty acid desaturase 2 (FAD2). (**J**) Sterol desaturase (SD). Relative expression of desaturase genes in endosperm was detected at different developmental stages. The gene transcripts were measured by qRT-PCR. Results are shown as the relative expression of genes at different developmental stages by comparing to itself at the highest expression, which was set as “1”. The experiments were performed in triplicate and the data are presented as means ± SD.

**Figure 5:**
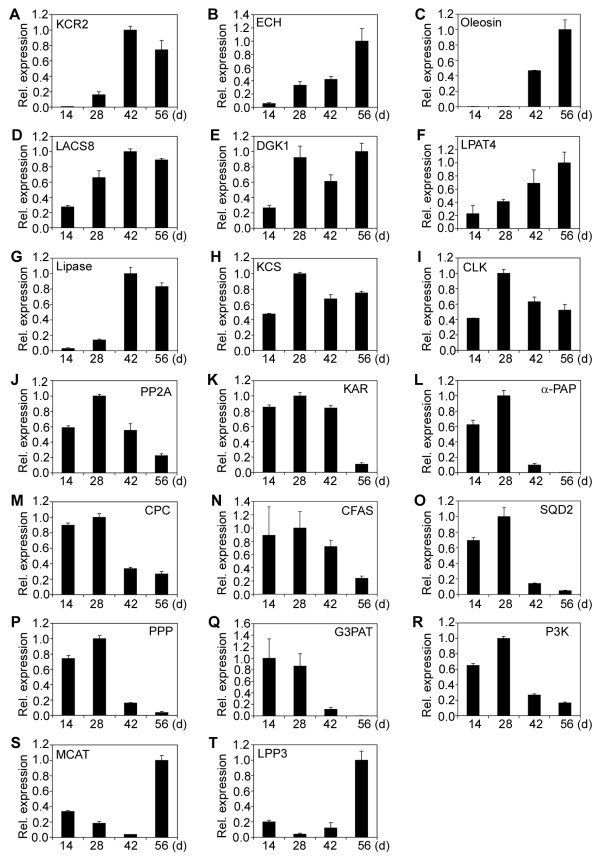
**Relative expression of fatty acid and lipid biosynthetic genes (III).** (**A**) 3-ketoacyl-CoA reductase 2 (KCR2). (**B**) enoyl-CoA hydratase (ECH). (**C**) Oleosin. (**D**) Long-chain acyl-CoA synthase 8 (LACS8). (**E**) Diacylglycerol kinase 1 (DGK1). (**F**) Lysophosphatidyl acyltransferase 4 (LPAT4). (**G**) Lipase. (**H**) Ketoacyl-CoA synthase (KCS). (**I**) Choline kinase (CLK). (**J**) Protein phosphatase 2A (PP2A). (**K**) Ketoacyl ACP reductase (KAR). (**L**) Phosphatidic aid phosphatase α (α-PAP). (**M**) Cholinephosphate cytidylyltransferase (CPC). (**N**) Cyclopropane-fatty-acyl-phospholipid synthase (CFAS). (**O**) Sulfoquinovosyldiacylglycerol synthase type-2 (SQD2). (**P**) Phosphotyrosine protein phosphatase (PPP). (**Q**) Glycerol-3-phosphate acyltransferase (G3PAT). (**R**) Phosphatidylinositol 3-kinase (P3K). (**S**) Malonyl CoA ACP transacylase (MCAT). (**T**) Lipid phosphate phosphatase 3 (LPP3). The gene transcripts were measured by qRT-PCR. Results are shown as the relative expression of genes at different developmental stages by comparing to itself at the highest expression, which was set as “1”. The experiments were performed in triplicate and the data are presented as means ± SD.

A normalized cDNA library of jatropha developing endosperm was constructed in our previous study [14]. Sixty-eight genes encoding enzymes, proteins or their subunits involved in fatty acid and lipid biosynthesis were identified. They were characterized for gene expression by qRT-PCR. Based on patterns of relative expression of genes at different developmental stages, the sixty-eight genes could be divided into five categories (Category I to Category V) (Table 1). Category I has 47 genes and they displayed a bell-shaped pattern of expression, which had peak expression at 28 or 42 DAF, but low expression at 14 and 56 DAF (Figure 2; Figure 3; Figure 4A to 4I). Category II comprises of 8 genes and they showed a constant increase in gene expression from 14 to 56 DAF (Figure 4J; Figure 5A to 5G). Category III contains 2 genes and both genes were constitutively expressed throughout endosperm development (Figure 5H and 5I). Category IV includes 9 genes and all of them displayed a high expression at 14 and 28 DAF, but a decreased expression at 42 and 56 DAF (Figure 5J to 5R). Finally, Category V has two genes and both genes showed a medium expression at 14 DAF, the lowest expression at 28 or 42 DAF and the highest expression at 56 DAF (Figure 5S and 5T).

**Table 1:** Summary of fatty acid and lipid biosynthetic genes in this study

**Gene**	**Category**^**1**^	**Forward primer (5’–3’)**	**Reverse primer (5’–3’)**	**Accession no.**
Ketoacyl ACP synthase I (KAS I)	I	TGGTTGGTCTTCTTTCCCTC	ACAAACCCACCAGCTTCATAG	JQ806261
Ketoacyl ACP synthase II (KAS II)	I	ACAGTCACCTTGTTTTTGTTCC	ATGCTAATTTACCCTGATAAGG	JQ806262
Ketoacyl ACP synthase III (KAS III)	I	CAATTATTAGATGGGGCTGAAG	TATGTGACAACAGAAACCAAGC	JQ806263
3-ketoacyl-CoA reductase isoform 1 (KCR1)	I	TCCTTTATTTGGGGTTTGTTAC	AGCAACCTAAAAGTTCAATTCC	JQ806264
3-ketoacyl-CoA reductase isoform 3 (KCR3)	I	ATGGACAGTAACATAGCCAATC	TCCACACACTTTTTCACAACTG	JQ806266
Acyl carrier protein1 (ACP1)	I	ACCGTTCAGGAAGCTGCTG	ACATAGTTCACAACAAGATTGC	JQ806272
Acyl-CoA dehydrogenase (ACD)	I	AATTGCAGATGGCCCCGATG	AGTTTTTGTGTGCTGGTATTGG	JQ806273
Semialdehyde decarboxylase 1 (SAD1)	I	GGGGCAGTGATTTTCCATTTG	GTGCTTTTTCCATATCTAATGAC	JQ806274
Hydroxyacyl-ACP dehydratase (HAD)	I	CTGCTATCCTATGCCTTTTTTG	GTTTTGCCCATAAGTTTAACATC	JQ806275
Acyl-ACP thioesterase (FATA)	I	TATTTGTGTGGGCATCTGCC	TAGTTGGTAAGGTGGGTTTAAC	JQ806276
Enoyl-ACP reductase (EAR)	I	ATGGGTGTGGGAGTTGACAG	GTGACATGGCATGGATTAAATG	JQ806277
Phosphatidic acid phosphatase β (β-PAP)	I	CACGAGCCCCATTCTGGAC	AGTCTGTTCAAGGTCAGGGG	JQ806281
CDP-diacylglycerol synthase (CDP-DAG)	I	CTAATAACAGTGTCATGGCAG	TTCCATATTCACTAAGTGCATTG	JQ806282
Digalactosyldiacylglycerol synthase 1 (DGD1)	I	AGACCTGCATCTCTACCTCC	GGTGCTGCCTAAATCTATATTC	JQ806283
Monogalactosyldiacylglycerol synthase (MGD2)	I	GTGTAAAGAATGGCAAGCATG	CCCCTAAAAGAATCAGAAACC	JQ806284
Phosphatidylinositol synthase (PTS)	I	CTTTTCATCTTCTGTGTCCATG	TAGTCAATAACCATCTCGTGC	JQ806287
Short-chain acyl-CoA oxidase (SCAOX)	I	AGGTTCTGCTTTTGCGCTAC	GGTCCCCTAGCTGGTAATTC	JQ806289
Long-chain acyl-CoA oxidase (LCAOX)	I	TGCACCAAGAGTATGATAGGC	TTACGTTTCTTTGTTCCAGCC	JQ806290
Acyl-CoA oxidase (AOX)	I	CCGTAATGCAAGACTGTGAAG	TTGCCATTAACTTGGATACAGC	JQ806291
Long-chain acyl-CoA synthetase (LACS)	I	CAAGAGAGAGGCCATCAGG	AAAATCCAAGAGAAACAGCAAG	JQ806292
Oleosin 3 (Oleosin 3)	I	AAGAGAAGTGGGTTTTGGTGG	AGAAACAAAAAGATTTAAGCG	JQ806304
Diacylglycerol kinase (DGK)	I	GTGGCTCAGATTTGGGTTGC	AAACTATTGAAGCTAAGCCTGG	JQ806306
D-erythro-sphingosine kinase/diacylglycerol kinase (DeDGK)	I	ATCAAATTCAGGAAAAGTAGCG	AATCAAACTGCACAAAAGGAAC	JQ806308
Calmodulin-binding diacylglycerol kinase (cDGK)	I	AGAAGATAAGGAAGAGCGAAG	GTTATAGCCTACAGCCAAAGC	JQ806309
Protein phosphatase 2C (PP2C)	I	TCATGGGCTTAAATGTGTGTAC	AACTCATACTTGAAAAGCTAAGG	JQ806311
Tyrosine phosphatase (TP)	I	TGGCCGTTTGTTAAG ATTGATC	ATTGACTTCATAATGTTGACCC	JQ806312
Phosphoglycolate phosphatase (PGP)	I	ATGCTGATGGGCTTTACTTTG	AGAACTAGCAAACTCCTTCCC	JQ806314
Lysophosphatidyl acyltransferase 1(LPAT1)	I	CTGAAGGTTAGTGCAACAAATG	GTAACATCGTCTGGAAAATTGC	JQ806317
Lysophosphatidyl acyltransferase 2 (LPAT2)	I	CTTTGGTTTCATGTGCTGCAC	ATACATGAAAAGAAAAGGTGCC	JQ806320
Lysophosphatidyl acyltransferase 5 (LPAT5)	I	TTTGGCATCTGCAACCTATTTC	GCCATAAACAGGTATGAGTCTC	JQ806319
Diacylglycerol acyltransferase (DGAT1)	I	GACCTAATGAATCGGAAAGGC	CCGCATAGCCAAAATTGCTTG	JQ806316
Phospholipid/glycerol acyltransferase (PL/GA)	I	TAAAGTATTCTCGCCCTAGCCC	ACATTTGCTTCTGTTTTCATGC	JQ806322
Triacylglycerol lipase (TAGL)	I	GTGAATACTGTTGTAAGCCTG	GTCCAAAAACACCAATGAAATG	JQ806324
Phospholipase C (PLC)	I	CAGCTCAATGGTGATTATGTC	AGCTTTTATGTAATTTGCGTCG	JQ806325
Phospholipase D (PLD)	I	ACTATGGGCAGAGCATGTTG	TCACATCCAGGAATAGCCTC	JQ806326
Phospholipase D α (PLDα)	I	CGCCAAATCTGATTACCTCC	CACTGATATGAACATCCTGGC	JQ806327
Phosphatidylinositol 4-kinase (P4K)	I	AAGACTTCTAGGGTTTGTGGG	CTCCTCAGTCCTCACTTAGC	JQ806329
Putative phosphatase (PP)	I	TGGGAAGATGCCATGTCTATC	CCAAACAAGAGATAAACTAACAG	JQ806330
Fatty acid desaturase (FAD)	I	ATGACCAATCCTGTTCCAAAG	TGCTAATGTTTACAAATGAGGG	JQ806294
Fatty acid desaturase 5 (FAD5)	I	ACTTGGTATGTTGTGAGGTTTC	ATGTAGAAAAGCTAATGCCCC	JQ806295
Fatty acid desaturase 6 (FAD6)	I	TTGCCCCTGAAGAATCTCAAC	ATTCATATTACTGTCCTCCCC	JQ806296
Acyl-ACP desaturase (AAD)	I	AGTTTTTGATCGGACGGTGG	AAAGAGAAGAAAGCAAGACTCG	JQ806300
δ-12-acyl-lipid desaturase (DALD)	I	GCTATAATATGTGGTTTGGCC	GTTGTAGAGTTCCATAAACGG	JQ806301
δ-7-C-5 sterol desaturase (D7SD)	I	TCTTGCATAGGCATCCATTTC	AGATCGAGTACATGGCTATGG	JQ806302
δ-9-stearoyl-acyl carrier protein desaturase (D9SD)	I	TGTTTGGAGAAGACATACCGG	CAGGGCTGTGGTGACTTAC	JQ806303
δ-12 fatty acid conjugase (D12FAC)	I	AGCCAGAAGAGGGAGGTCC	AACTCAAACACCACTTTCCCG	JQ806298
Fatty acid desaturase 2 (FAD2)	I	TGGACTACTTGTTAGGAATTTG	GTTCATATTGTTTCTACCTGGG	JQ806297
Sterol desaturase (SD)	II	CTGATGAAGGCACACTGTTG	GTAGAAACATAATCCACTGCC	JQ806299
3-ketoacyl-CoA reductase isoform 2 (KCR2)	II	CGTCTCTCTCTCGGAATCC	TCTTCTGTGAAAACGACCCTC	JQ806265
Enoyl-CoA hydratase (ECH)	II	AGATTGGAGGAATGGTCAAAG	TATTGCTTGCTAGGATTGGAG	JQ806267
Oleosin (Oleosin)	II	TGGACAGTATATGCAACACAAG	TATTCCACACTGAAATTAGCAC	JQ806305
Long-chain acyl-CoA synthetase 8 (LACS8)	II	TTTGTTTAATGTGCTTTCCTCC	GTCCTGCAATTTAGGTGAAGC	JQ806293
Diacylglycerol kinase 1 (DGK1)	II	TGGCACTTAGGCTGACTTAG	CAGCTAAAAGCACCAAGTTAAG	JQ806307
Lysophosphatidyl acyltransferase 4 (LPAT4)	II	ATTTTAGCATGTGCATTCCTTG	TATAAACAAGTTCACAAAAAGGTC	JQ806318
Lipase (Lipase)	II	TGGGGTTCAATGCCAAAGAC	TAGCCTGTCTACAGATTTTCC	JQ806323
Ketoacyl-CoA synthase (KCS)	III	CTTTGATTTGTACTTTCATGGG	AACACACAAGCATTTGAAGCC	JQ806268
Choline kinase (CLK)	III	TATTTCTTCCTGCGATACAATG	TTTGGATCTTAAATCTGGCTAC	JQ806313
Protein phosphatase 2A (PP2A)	IV	TGATGTTACCCGTAGAACTCC	ATAAAAAAAAACAAAACCTGCCC	JQ806310
Ketoacyl ACP reductase (KAR)	IV	CGGAAGAGGTTGCAGGATTG	AAAAACTGCCTCAACACAAGC	JQ806278
Phosphatidic acid phosphatase α (α-PAP)	IV	ATGGGGCTATATTTGGCTCAC	CTTGGAAACCTGATAAACAAAAG	JQ806280
Cholinephosphate cytidylyltransferase (CPC)	IV	GACAAAGATGATGCTAAGGAG	TATTCCTATCCTCACAACAAGC	JQ806285
Cyclopropane-fatty-acyl-phospholipid synthase (CFAS)	IV	GACTTGTCTTCCTGAGAGCC	GCAGTTCTTTGAAAGCGATGG	JQ806286
Sulfoquinovosyldiacylglycerol synthase type 2 (SQD2)	IV	GCAGCCACTAGAAAAATCCG	GCAGAGCAAATCCGTCACC	JQ806288
Phosphotyrosine protein phosphatase (PPP)	IV	ATGTGCTCCTTTTGTAAGAAAC	AACTTTGAATGCCGCTGGTC	JQ806315
Glycerol-3-phosphate acyltransferase (G3PAT)	IV	GGCAGTAATGTCTTTGGTTTC	TACATGAAAAGAAAGGGTGCC	JQ806321
Phosphatidylinositol 3-kinase (P3K)	IV	TGGAGTTTGCTCAAGGAGTC	TTAAAAAAGAGCTGAAAGACACC	JQ806328
Malonyl CoA ACP transacylase (MCAT)	V	GTTATTGCTGGCATTGTCAAG	GAAATCTCTAGTACATGACGC	JQ806279
Lipid phosphate phosphatase 3 (LPP3)	V	TCTGAGACGAGCGGAGGAC	ATTCATCATCTCCTTCCAGTTC	JQ806270
Jatropha Actin 1 protein (Actin1)	Control	TAATGGTCCCTCTGGATGTG	AGAAAAGAAAAGAAAAAAGCAGC	JQ806331

^1^The genes can be grouped into five categories based on their expression patterns during endosperm development. Please refer to the text for details.

The 47 genes of Category I encode ketoacyl ACP synthase I (KAS I) (Figure 2A), ketoacyl ACP synthase II (KAS II) (Figure 2B), ketoacyl ACP synthase III (KAS III) (Figure 2C), 3-ketoacyl-CoA reductase isoform 1 (KCR1) (Figure 2D), 3-ketoacyl-CoA reductase isoform 3 (KCR3) (Figure 2E), plastial acyl carrier protein isoform 1 (ACP1) (Figure 2F), acyl-CoA dehydrogenase (ACD) (Figure 2G), semialdehyde decarboxylase 1 (SAD1) (Figure 2H), hydroxyacyl-ACP dehydratase (HAD) (Figure 2I), acyl-ACP thioesterase (FATA) (Figure 2J), enoyl-ACP reductase (EAR) (Figure 2K), phosphatidic acid phosphatase β (β-PAP) (Figure 2L),CDP-diacylglycerol synthase (CDP-DAG) (Figure 2), digalactosyldiacylglycerol synthase 1 (DGD1) (Figure 2N), monogalactosyldiacylglycerol synthase (MGD2) (Figure 2O), phosphatidylinositol synthase (PTS) (Figure 2P), short-chain acyl-CoA oxidase (SCAOX) (Figure 2Q), long-chain acyl-CoA oxidase (LCAOX) (Figure 2R), acyl-CoA oxidase (AOX) (Figure 2S), long chain acyl-CoA synthetase (LACS) (Figure 2T), Oleosin 3 (Figure 3A), diacylglycerol kinase (DGK) (Figure 3B), D-erythro-sphingosine kinase/diacylglycerol kinase (DeDGK) (Figure 3C), calmodulin-binding diacylglycerol kinase (cDGK) (Figure 3D), protein phosphatase 2C (PP2C) (Figure 3E), tyrosine phosphatase (TP) (Figure 3F), phosphoglycolate phosphatase (PGP) (Figure 3G), lysophosphatidyl acyltransferase 1 (LPAT1) (Figure 3H), lysophosphatidyl acyltransferase 2 (LPAT2) (Figure 3I), lysophosphatidyl acyltransferase 5 (LPAT5) (Figure 3J), diacylglycerol acyltransferase (DGAT1) (Figure 3K), phospholipid/glycerol acyltransferase (PL/GA) (Figure 3L), triacylglycerol lipase (TAGL) (Figure 3M), phospholipase C (PLC) (Figure 3N), phospholipase D (PLD) (Figure 3O), phospholipase D α (PLDα) (Figure 3P), phosphatidylinositol 4-kinase (P4K) (Figure 3Q) and putative phosphatase (PP) (Figure 3R), fatty acid desaturases (FAD) (Figure 4A), δ-9 fatty acid desaturase (FAD5) (Figure 4B), chloroplast omega-6 fatty acid desaturase 6 (FAD6) (Figure 4C), acyl-ACP desaturase (AAD) (Figure 4D), δ-12-acyl-lipid desaturase (DALD) (Figure 4E), δ-7-C-5 sterol desaturase (D7SD) (Figure 4F), δ-9-stearoyl-acyl carrier protein desaturase (D9SD) (Figure 4G), δ-12 fatty acid conjugase (D12FAC) (Figure 4H) and δ-12-fatty acid desaturase (FAD2) (Figure 4I), respectively.

The 8 genes of Category II encode sterol desaturase (SD) (Figure 4J), 3-ketoacyl-CoA reductase isoform 2 (KCR2) (Figure 5A), enoyl-CoA hydratase (ECH) (Figure 5B), Oleosin (Figure 5C), long-chain acyl-CoA synthetase 8 (LACS8) (Figure 5D), diacylglycerol kinase 1 (DGK1) (Figure 5E), lysophosphatidyl acyltransferase 4 (LPAT4) (Figure 5F) and lipase (Lipase) (Figure 5G), respectively. The 2 genes of Category III encode ketoacyl-CoA synthase (KCS) (Figure 5H) and choline kinase (CLK) (Figure 5I), respectively. The 9 genes of Category IV encode protein phosphatase 2A (PP2A) (Figure 5J), ketoacyl ACP reductase (KAR) (Figure 5K), phosphatidic acid phosphatase α (α-PAP) (Figure 5L), cholinephosphate cytidylyltransferase (CPC) (Figure 5M), cyclopropane-fatty-acyl-phospholipid synthase (CFAS) (Figure 5N), sulfoquinovosyldiacylglycerol synthase type 2 (SQD2) (Figure 5O), phosphotyrosine protein phosphatase (PPP) (Figure 5P), glycerol-3-phosphate acyltransferase (G3PAT) (Figure 5Q) and phosphatidylinositol 3-kinase (P3K) (Figure 5R), respectively. Finally, the 2 genes of Category V encode malonyl CoA ACP transacylase (MCAT) (Figure 5S) and lipid phosphate phosphatase 3 (LPP3) (Figure 5T), respectively.

### Differential expression of genes encoding enzymes or proteins with similar function in fatty acid and lipid biosynthesis

**Figure 6:**
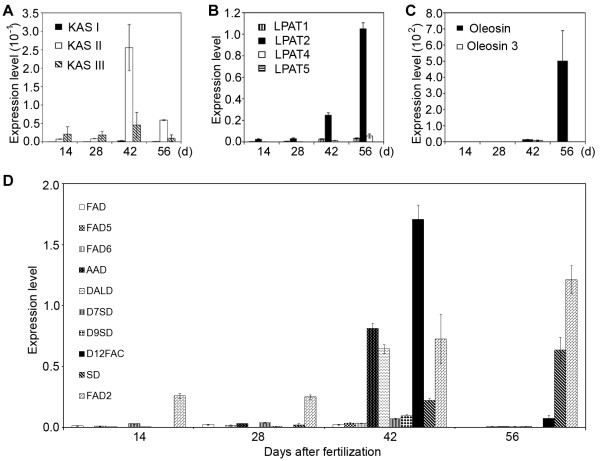
**Differential expression of genes encoding enzymes with similar function in fatty acid and lipid biosynthesis.** (**A**) Expression of ketoacyl ACP synthase (KAS) genes. KAS I, ketoacyl ACP synthase I; KAS II, ketoacyl ACP synthase II; KAS III, ketoacyl ACP synthase III. (**B**) Expression of lysophosphatidyl acyltransferase (LPAT) genes. LPAT1, Lysophosphatidyl acyltransferase 1; LPAT2, Lysophosphatidyl acyltransferase 2; LPAT4, Lysophosphatidyl acyltransferase 4; LPAT5, Lysophosphatidyl acyltransferase 5. (**C**) Expression of oleosin genes. **(D)** Expression of desaturase genes. FAD, fatty acid desaturase; FAD5, fatty acid desaturase 5; FAD6, fatty acid desaturase 6; AAD, acyl-ACP desaturase; DALD, δ-12-acyl-lipid desaturase; D7SD, δ-7-C-5 sterol desaturase; D9SD, δ-9-stearoyl-acyl carrier protein desaturase; D12FAC, δ-12 fatty acid conjugase; SD, sterol desaturase; FAD2, fatty acid desaturase 2. The gene transcripts were measured by qRT-PCR. Results are shown as the ratios of transcripts fatty acid and lipid biosynthetic genes over that of the *Actin1* gene of jatropha. The experiments were performed in triplicate and the data are presented as means ± SD.

Differential expression of genes encoding enzymes or proteins with similar function in fatty acid and lipid biosynthesis were observed during endosperm development. To determine expression levels of these genes, we calculated the ratio of transcripts of each gene to that of jatropha Actin 1 gene at different developmental stages. Figure 6 shows the differential expression of genes that encode ketoacyl ACP synthases (Figure 6A), lysophosphatidyl acyltransferases (Figure 6B), oleosin proteins (Figure 6C) and desaturases (Figure 6D), respectively, at different endosperm developmental stages. Although all of the three KAS genes showed an expression pattern of Category I, they were differentially expressed at different developmental stages. The ratio of transcripts of KAS I:KAS II:KAS III was 1:92:16 at 42 DAF, whereas the ratio became 1:71:12 at 56 DAF (Figure 6A). Four LPAT genes were identified to be expressed in jatropha developing endosperm. The LPAT1, LPAT2 and LPAT5 genes showed an expression pattern of Category I with the highest expression at 28 DAF (Figure 3H to 3J), whereas the LPAT4 gene displayed an expression pattern of Category II with gene expression constantly increased from 14 to 56 DAF (Figure 5F). However, at both 42 and 56 DAF, the majority of transcripts were expressed from the LPAT2 gene (Figure 6B). The Oleosin gene showed peak expression at 56 DAF (Category II) (Figure 5C), whereas the Oleosin 3 gene displayed maximal expression at 42 DAF (Category I) (Figure 3A). The ratio of transcripts of Oleosin:Oleosin 3 was 1.7:1 at 42 DAF, whereas it was drastically increased to 3593:1 (Figure 6C), indicating that the Oleosin gene was the major gene encoding oleosin proteins for oil body formation at the late stages of endosperm development.

Ten desaturase genes were identified to be expressed in jatropha developing endosperm (Figure 4). Nine of them, including the FAD, FAD5, FAD6, AAD, DALD, D7SD, D9SD, D12FAC and FAD2 genes, showed an expression pattern of Category I and only the SD gene displayed an expression pattern of Category II (Figure 4). The FAD2 gene had a peak expression at 28 DAF (Figure 4I). Although the gene was assigned to Category I, it was also highly expressed at 14 and 56 DAF, respectively (Figure 4I). Actually, the FAD2 gene was the most highly expressed gene at 14, 28 and 56 DAF among the 10 desaturase genes and one of the highly expressed genes at 42 DAF (Figure 6D). The results demonstrated that FAD2-mediated fatty acid desaturation is required for lipid biosynthesis involved in both endosperm development and storage lipid accumulation. At 42 DAF, the transcripts from the AAD, DALD, D12FAC and FAD2 genes occupied 90% of the total transcripts derived from all desaturase genes and the D12FAC gene alone contributed 40% of the total transcripts (Figure 6D). In addition, unlike other desaturase genes, both DALD and D12FAC genes were maximally expressed at 42 DAF (Figure 4E and 4H). However, their transcripts were almost undetectable at 14, 28 or 56 DAF (Figure 4E and 4H). The SD gene showed an expression pattern of Category II and had a peak expression at 56 DAF (Figure 4J). Its transcripts contributed 30% of the total transcripts derived from all desaturase genes detected (Figure 6D). Based on the results, the AAD, DALD, D12FAC, FAD2 and SD genes are major desaturase genes that are involved in storage lipid biosynthesis in jatropha endosperm.

## Discussion

TEM studies indicate that storage lipids in jatropha developing endosperm are synthesized from 28 to 56 DAF. Consistent with this observation, almost all of the 68 genes identified in this study showed high or peak expression at either one or two or even three stages from 28 to 56 DAF. Genes with expression patterns of Categories I, II and IV may encode the core enzymes or proteins or their subunits that are required for storage lipid biosynthesis. Genes with expression patterns of Categories III and IV may be involved in biosynthesis of fatty acid and lipids or lipid signaling, which are essential for endosperm development. The analysis on differential expression of genes that encode enzymes or proteins with similar function in fatty acid and lipid biosynthesis may provide clues to identify these key genes that play pivotal roles in the limiting steps of storage lipid biosynthesis. The information on gene expression levels and patterns provides guideline on genetic breeding and genetic engineering of jatropha for increasing oil content or changing profiles of fatty acid and lipids in jatropha seeds.

KAS is involved in the formation of acetoacetyl ACP. All plants examined to date contain three KAS isoenzymes (I, II, and III) and each distinguishes by its substrate specificity [15]. Our studies demonstrated that all three KAS genes showed an expression pattern of Category I (Figure 2A to 2C). The KAS I and KAS III genes showed peak expression at 28 DAF, whereas the KAS II gene was maximally expressed at 42 DAF (Figure 2A to 2C). The latter result was slightly different from a previous study, in which the KAS II gene showed a peak expression at 50 DAP (days after pollination), when the jatropha seeds were almost fully matured [12].

KCS is a component of the elongation complex responsible for the synthesis of very long chains of monounsaturated fatty acids (VLCMFA) in the seeds of plants [16]. The KCS gene showed an expression pattern of Category III, which was constitutively expressed throughout endosperm development (Figure 5H). Our result was similar to that of *FAE1* or *KCS* gene in *Brassica napus*[17]. The results indicate that KCS, which determines fatty acid profiles in storage lipids, is not regulated at the transcription level. Taylor et al. (2009) produced transgenic Arabidopsis and *Brassica Carinata* plants that expressed Cardamine KCS gene. The seed-specific expression of the Cardamine KCS gene led to 55-fold and 15-fold increase in nervonic acid proportions in Arabidopsis and *B. carinata* seed oil, respectively [16].

FATA is a intraplastidial enzyme that terminates the synthesis of fatty acids in plants [18]. It also facilitates the export of acyl moieties to endoplasmic reticulum where they can be used in the production of glycerolipids [18]. The FATA gene showed gene expression of Category I in jatropha developing endosperm (Figure 2J). In Arabidopsis FATA mutant, palmitate (16:0) and stearate (18:0) contents were reduced to 56% and 30 % in seeds, suggesting that FATA plays a major role in determining the types of fatty acids. Analysis of individual glycerollipids revealed a 4-fold reduction of 16:0 and a 10-fold reduction of 18:0 in the FATA mutant [19]. Further analysis showed that FATA is involved in biosynthesis of saturated fatty acids, which are essential for plant growth and development [19].

The phospholipid biosynthetic enzyme, LPAT, catalyzes the acylation of lysophosphatidic acid to form phosphatidic acid, the major precursor of all glycerolipids [20]. Four LPAT genes were identified in this study. The LPAT1, LPAT2 and LPAT5 genes showed a expression pattern of Category I, whereas the LPAT4 gene displayed an expression pattern of Category II, whose expression was constantly increased from 14 to 56 DAF (Figure 3H to 3J; Figure 5F). These results support the hypothesis that increasing the expression of glycerolipid acyltransferase in seeds leads to a greater flux of intermediates through the Kennedy pathway and enhanced triacylglycerol accumulation [21]. Indeed, overexpression of two rapeseed LPAAT (LPAT) isozymes in Arabidopsis increased lipid content and seed mass in seeds [21]. Considering the LPAT2 gene is the only LPAT gene that is highly expressed at both 42 and 56 DAF (Figure 6B), it may be used to be overexpressed in jatropha endosperm at late developmental stages to enhance storage lipid production.

DGAT catalyzes the final step of lipid synthesis in many plants. Its expression level is correlated with lipid accumulation. The DGAT1 gene in jatropha showed an expression pattern of Category I, which displayed high expressions at 28 and 42 DAF and a decreased expression at 56 DAF (Figure 3K). Previous studies have shown that a phenylalanine insertion in DGAT1-2 at position 469 (F469) is responsible for the increased oil and oleic-acid contents in maize [22]. As one of the oil quantitative trait loci (QTLs), ectopic expression of the high-oil *DGAT1-2* allele increases oil and oleic-acid contents up to 41 % and 107 %, respectively [22]. The DGAT activity in developing seeds of transgenic lines was enhanced by 10 % to 70 % [22]. In addition, overexpression of a diacylglycerol acyltransferase 2A from soil fungus *Umbelopsis ramanniana* in soybean seed led to a 1.5 % increase in oil yield in the mature seed [23]. Based on these reports, overexpression of the DGAT gene in transgenic jatropha plants may have high potential to increase the oil yield.

Desaturases play a pivotal role in fatty acid desaturation during fatty acid and lipid biosynthesis. Ten desaturase genes were identified to be expressed in developing jatropha endosperm. Most of the desaturase genes showed an expression pattern of Category I except that the SD gene displayed an expression pattern of Category II (Figure 4). The expression pattern of the FAD6 gene in this study, which had a peak expression at 28 DAF, was different from a previous study, in which chloroplast-6 fatty acid desaturase (Chlo 6 or FAD6) gene showed the maximum expression at 50 DAP [12]. The desaturase genes are good candidates for engineering oil plants to increase or decrease the production of polyunsaturated fatty acids. Recent study demonstrated that downregulation of *JcFAD2-1* in jatropha by RNA interference technology caused a dramatic increase of oleic acid (> 78 %) and a corresponding reduction in polyunsaturated fatty acids (< 3 %) in its seed oil [24]. The AAD, DALD, D12FAC and FAD2 genes were the major desaturase genes that were highly expressed at 42 DAF (Figure 6D). Likewise, both SD and FAD2 genes were the major desaturase genes that were highly expressed at 56 DAF (Figure 6D). These desaturase genes are potential candidates for genetic engineering to modify polyunsaturated fatty acids in jatropha seed oil.

In plants, storage lipds are generally stored in oil body that is enclosed with a single layer of phospholipid rich in oleosin proteins. Seeds with high oil content have more oleosins than those with low oil content [25]. The exact role of oleosin in oil accumulation is unclear, although it may be involved in the biosynthesis and mobilization of plant oils. Previous study demonstrated that the relative net amounts of oleosin and oil accumulation during seed development are the major determinants of oil-body size in desiccation-tolerant seeds [26]. Xu et al. (2011) found that *Ole1* and *Ole2* showed maximum expression at 50 DAF [12]. Two oleosin genes, the Oleosin and Oleosin 3 genes, were identified in this study. The Oleosin gene showed an expression pattern of Category II (Figure 5C), whereas the Oleosin 3 genes displayed an expression pattern of Category I (Figure 3A). More importantly, the Oleosin gene was the major oleosin gene that was expressed at the late stages of endosperm development (Figure 6C). In this scenario, over-expression of the Oleosin gene in developing jatropha endosperm, especially at the late stage, may have potential to increase oil yield in jatropha seeds.

## Conclusion

The formation of oil bodies in jatropha endosperm is developmentally regulated. The expression of most of the fatty acid and lipid biosynthetic genes is highly consistent with the development of oil bodies and endosperm in jatropha seeds, while the genes encoding enzymes or proteins with similar function may be differentially expressed during endosperm development. These results not only provide the initial information on spatial and temporal expression of fatty acid and lipid biosynthetic genes in jatropha developing endosperm, but also are valuable to identify the rate-limiting genes for genetic engineering of storage lipid biosynthesis and accumulation during seed development.

## Methods

### Plant material and plant growth condition

Jatropha plants were grown in the experimental field of Temasek Life Sciences Laboratory, Singapore, under natural climate conditions at a temperature of 30°C for 12.5 hr (light) and 22°C for 11.5 hr (dark). Flowers were manually pollinated and fruits and seeds from 14, 28, 42 and 56 DAF were harvested in liquid nitrogen, dissected and stored at −80°C for RNA extraction.

### Transmission electron microscopy

Tissues from developing seeds were fixed overnight in 2.5 % glutaraldehyde in 0.1 M phosphate buffer, pH 7.2. After being briefly rinsed in the buffer, samples were post-fixed for 2 h with 1 % osmium tetroxide in 0.1 M phosphate buffer, pH7.2. Samples were dehydrated through a graded series of ethanol before being embedded in Spurr’s resin. Ultrathin sections (90 nm) were cut with a diamond knife on an ultramicrotome (Leica Ultracut UCT; Leica, Germany) and mounted on 300-mesh copper grids. They were then stained with uranyl acetate and lead citrate, and examined with a transmission electron microscope (JEM-1230; JEOL, Japan) at 120 kV. Photographs were taken with a digital microphotography system (Gatan, USA).

### Sequencing of cDNA clones

The construction of normalized cDNA library and sequencing of cDNA clones were described previously [14]. Jatropha endosperm at 14 to 56 DAF were ground to fine powder in liquid nitrogen and total RNA was extracted using RNeasy Plant Mini Kit (Qiagen, Hilden, Germany) [27] for cDNA library construction. The first strand cDNA was generated with PowerScript Reverse Transcriptase (BD Biosciences Clontech) and primers SMART-Sfi1A oligonucleotides 5’-AAGCAGTGGTATCAACGCAGAGTGGCCATTACGGCCGGG-3’ and CDS-Sfi1B primer 5’-AAGCAGTGGTATCAACGCAGAGTGGCCGAGGCGGCCTTTTTTTTTTTTTTTTTTTT-3’. Ds cDNAs were prepared by using Long-Distance PCR (Barnes, 1994) and SMART PCR primer 5’-AAGCAGTGGTATCAACGCAGAGT-3’. Colony PCR was conducted with primers pAL17 dir: 5’-CCAGGGTTTTCCCAGTCACGA-3’ and pAL17 rev: 5’-CACAGGAAACAGCTATGACCA-3’. The chromatograms from the data set were processed using Sequencher v3.10 (Gene Codes, Ann Arbor, MI). Sequences that were less than 100 bp long or had 4 % ambiguities were not processed.

### Real-time PCR analysis

Total RNA was extracted from jatropha developing endosperm using standard procedures. The RNA samples were treated with DNase I and then used to synthesize first-strand cDNA using iScript cDNA synthesis kit (Bio-Rad). Specific primers were designed based on the DNA sequences at 3’ untranslation regions (3’UTR) for each gene (Table 1). To ensure maximum specificity and efficiency during quantitative PCR, putative primer pairs were tested for linearity of response by constructing standard curves on five or six serial ten-fold dilutions. A standard reaction mixture (15 μl) contained 2 μl cDNA templates, 1 x SsoFast EvaGreen supermix and 500 nM forward and reverse primers. The quantitative PCR analysis was conducted on a Bio-Rad iCycler iQ5 real-time PCR system. The PCR reaction consisted of an initial denaturizing step of 95°C for 30 sec, followed by 40 cycles of 95°C for 5 sec and finally 60°C for 10 sec. A melting-curve reaction immediately followed the amplification with heating for 10 sec, starting at 65°C with 0.5°C increments. The specificity of PCR product was confirmed by melting-curve analysis and electrophoresis on 2 % agarose gel to ensure that PCR reactions were free of primer dimmers. The jatropha *actin* gene was used as the internal control. For each gene, three repeated experiments, including internal controls and negative controls (reaction samples without cDNA templates), were conducted.

### Accession numbers

Partial cDNA sequences of jatropha genes have been submitted to GenBank at NCBI with the accession numbers JQ806261 to JQ806331.

## Competing interests

The authors declared that they have no competing interests.

## Authors’ contributions

K Gu and Z Yin designed experiments and analyzed experimental data. K Gu, S Jatinder and D Tian conducted the experiments. C Yi and Y Hong contributed jatropha developing seeds. K Gu and Z Yin co-wrote the manuscript. All authors read and approved the final manuscript.
